# The Spatial Dynamics of Japanese Sardine (*Sardinops sagax*) Fishing Grounds in the Northwest Pacific: A Geostatistical Approach

**DOI:** 10.3390/ani15111597

**Published:** 2025-05-29

**Authors:** Yongzheng Tang, Yuanting Gong, Heng Zhang, Guoqing Zhao, Fenghua Tang

**Affiliations:** 1 School of Ocean, Yantai University, Yantai 264005, China; 13906380063@163.com (Y.T.); 13371381944@163.com (Y.G.); 2 East China Sea Fisheries Research Institute, Chinese Academy of Fishery Sciences, Shanghai 200090, China; zhangziqian0601@163.com; 3 Key Laboratory of Oceanic and Polar Fisheries, Ministry of Agriculture and Rural Affairs, Beijing 100125, China

**Keywords:** *Sardinops sagax*, spatio-temporal variations, standard deviation ellipse, spatial autocorrelation, hotspot analysis, GAM, climate change

## Abstract

The Japanese sardine (*Sardinops sagax*), a key economic species in the Northwest Pacific Ocean (NWPO), has shown significant increases in both population abundance and catch volume over the past decade. To understand its spatiotemporal dynamics under climate change, this study analyzed light purse seine fishery data (2014–2021) from the NWPO. The results showed that the primary fishing season spans March to December, with peak catches concentrated in 40–43° N, 149–155° E. Annual catches grew steadily, accelerating notably in 2021. The fishing grounds’ center shifted northeastward annually and seasonally (southwest-to-northeast trajectory), driven by directional aggregation. Spatial clustering with global positive autocorrelation was observed, weakening as distance thresholds increased. Resource hotspots migrated northeast, narrowing from 40–42° N (2016) to 42–44° N (2017–2021), while coldspots showed complementary fluctuations. Generalized additive model (GAM) analysis revealed that the catch per unit effort (CPUE) of Japanese sardine in the high seas of the NWPO was governed by temporal–spatial drivers and multivariate environmental determinants. Analytical findings substantiate the significant climate-driven impacts on the spatiotemporal distribution and population dynamics of Japanese sardine. The non-stationary interannual and seasonal patterns of fishing grounds highlight the need for adaptive management strategies.

## Simple Summary

The Japanese sardine (*Sardinops sagax*), a key economic species in the Northwest Pacific Ocean (NWPO), has undergone substantial geographical displacement in terms of both resource distribution and fishing grounds under climate change pressures. Through geostatistical analyses incorporating spatial autocorrelation and standard deviational ellipse (SDE) modeling, this study quantified the spatiotemporal dynamics of sardine fishing grounds. The results revealed northeastward movement of the fishing grounds’ center of gravity, with strong spatial clustering, and hotspot expansion toward the northeast. Spatiotemporal factors and environmental parameters exert significant influences on Japanese sardine resources. Climate change critically impacts population dynamics and fishing ground distribution, highlighting its importance in ecosystem-based fishery management. These findings offer guidance for sustainable fishery practices.

## 1. Introduction

The high seas of the Northwest Pacific Ocean (NWPO) form a biologically rich marine area where the Kuroshio and Oyashio currents converge, creating highly productive fishing grounds through nutrient enrichment [1,2]. As the Food and Agriculture Organization of the United Nations (FAO) Fishing Area 61, this region has long been a major global fishing ground [3]. China (including Taiwan Province), Japan, Russia, and South Korea lead production in the North Pacific Ocean through the North Pacific Fisheries Commission (NPFC) organization for sustainable fishing [4]. Key commercial species include the chub mackerel (*Scomber japonicus* Houttuyn, 1782), Japanese sardine (*Sardinops sagax* Jenyns, 1842), neon flying squid (*Ommastrephes bartramii* Lesueur, 1821), and Pacific saury (*Cololabis saira* Brevoort, 1856) [5,6]. Japanese sardine catches have shown notable annual increases, particularly in light purse seine fisheries [5].

The Japanese sardine is a schooling, migratory pelagic fish inhabiting warm waters around Japan and the NWPO [7,8]. Not only are these fish prized for their tender and succulent flesh and rich nutrient content, but they also hold high commercial value, making them a favorite among consumers. Their population consists of two stocks: the smaller Tsushima Warm Current stock (East China Sea and Sea of Japan) and the larger Pacific stock (Kuroshio Current region) [9,10], the latter being both the study focus and the primary fishery target [8]. In NWPO waters, this species serves as a keystone in the Kuroshio ecosystem by consuming zooplankton while being prey for larger predators [11,12,13,14], thus functioning as an ecological energy conduit. 

Japanese sardine populations exhibit large biomass but significant fluctuations [8,15]. FAO and NPFC data show that catches exceeded one million tons in the 1970s, followed by rapid growth until a sharp decline in the 1990s. After remaining low until 2010, recent years have seen recovery to over one million tons. Similar boom–bust cycles occur in California, Peru, and Chile [16], with 20–30 year fluctuations observed twice in the last century [17,18]. These dynamics have led scientists to study driving factors including sea surface temperature (SST) changes [19], extreme weather [20], fishing pressure [21], management practices [17], density-dependent effects [22,23], recruitment failure [24], and non-stationary multi-driver impacts [8]. 

Research indicates sustainable exploitation potential for Japanese sardine stocks in the NWPO [25], with fishing pressure being a secondary factor in population changes [26]. Understanding fishing ground dynamics is crucial for stock assessment and fishery forecasting. Effective management requires identifying spatial distribution patterns, yet few studies have examined fishing ground variability. There is, thus, an urgent need to investigate long-term Japanese sardine fishing ground variations.

Fishing ground distribution reflects fish stock dynamics across space and time. Traditional assessment methods typically use arithmetic means from fishery surveys [27,28,29], which remain key for studying spatial–temporal patterns, particularly in offshore ecosystems. For pelagic stocks, scientists primarily analyze fisheries’ production data, employing methods like catch gravity centers and geostatistical interpolation to track seasonal shifts [5]. Models such as GAM [30] help examine correlations between fishing ground variability and environmental factors (e.g., SST, chlorophyll, currents, thermocline) [8,31]. As spatiotemporal phenomena, fishing ground changes are effectively analyzed through geostatistical methods, which have proven robust for assessments [32].

Geostatistical analysis, a spatial method based on regionalized variability theory, effectively describes spatially correlated ecological data [33]. Fish distributions exhibit strong spatial autocorrelation and heterogeneity [34]. Unlike classical statistics that assume data independence [35,36], geostatistics enables quantitative analysis of fishery resource distributions [37,38]. This approach characterizes both density patterns and populations’ spatial structures [35,39], supporting stock assessment and fishery management. Current applications include hotspot identification [40,41], catch per unit effort (CPUE) standardization [34], distribution heterogeneity studies [33,42], seasonal variation analysis [43], and resource assessments [39]. 

The aims of this study are to understand the dynamics of Japanese sardine fishing grounds in the high seas of the NWPO and the key factors affecting the distribution of sardine fishing grounds, and to provide a scientific basis for research on the population dynamics, stock assessment, fishing production, and fishery management of Japanese sardine in the high seas of the NWPO.

## 2. Materials and Methods

### 2.1. Data Resources

**Figure 1:**
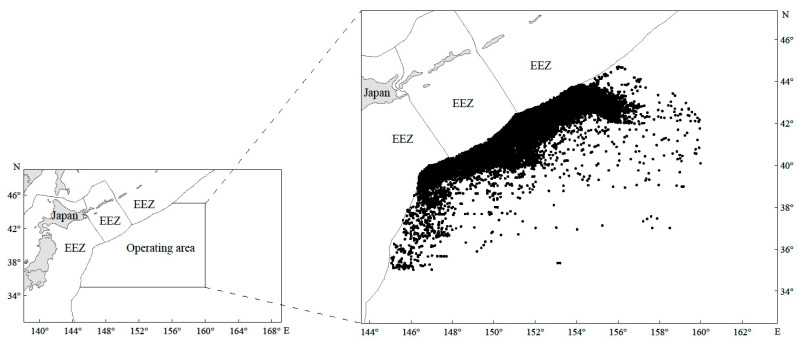
The distribution of Japanese sardine fishing locations (black spots) in the high seas of the NWPO from 2014 to 2021 (EEZ means exclusive economic zone).

The fishery data analyzed in this study were obtained from the Technical Group for Trawl–Purse Seine Fishery, China Distant–Water Fisheries Association, spanning 2014 to 2021 (*n* = 37,684 records). The main fishing time was from March to December each year, and the distribution of the study area was between 35–45° N and 145–160° E (Figure 1). The dataset includes the start and end time, yield, latitude, and longitude for each fishing net deployment. In this paper, 0.1° × 0.1° is defined as the unit fishing area when analyzing hotspot and coldspot areas, as well as GAM analysis, and the data within the unit fishing area have been converted to CPUE, which is used to represent the resource density of the Japanese sardine in the NWPO. The CPUE was calculated using the formula CPUE = C/E, where C represents the total catch (in tons) and E denotes the corresponding fishing effort, expressed as the number of fishing nets used [44].

The study identified SST, sea surface height (SSH), salinity (SSS), and chlorophyll-a (Chla) as the key environmental factors influencing Japanese sardine population dynamics [45]. These variables were consequently chosen as GAM parameters. The corresponding datasets were obtained from the Copernicus Marine Service (http://marine.copernicus.eu, accessed on 23 May 2023). All environmental data were obtained at a spatial resolution of 0.083° and a monthly temporal resolution. 

The geographic distribution maps and spatial autocorrelation maps in this study were created using ArcGIS 10.6 software.

### 2.2. Global Spatial Autocorrelation

Spatial autocorrelation refers to the potential dependence between observed elements within a given distribution area [46]. Global spatial autocorrelation describes the spatial characteristics of geographic attribute element values across the region and can measure the degree of association between spatial objects across the study area to indicate whether there is a significant spatial distribution pattern between spatial objects, which is usually analyzed using statistics such as the global Moran’s *I*, the global Gear’s C, and global statistics [47]. Moran’s *I* is a statistical measure used to assess spatial autocorrelation, primarily employed to analyze whether geographic data exhibit clustering or dispersion patterns in space. This study used the global Moran’s *I* to assess spatial autocorrelation, with significance tested via the standardized *Z*-value (calculated below) [48]:

(1) $$I=\frac{n\sum_{i=1}^{n}\sum_{j=1}^{m}w_{ij}\left(x_{i}-x\right)\left(x_{j}-x\right)}{\sum_{i=1}^{n}\sum_{j=1}^{m}w_{ij}{\left(x_{i}-x\right)}^{2}}(i\neq j)$$

(2) $${\;Z}_{score}=\frac{\;I\;-\;E(I)}{\sqrt{VAR(I)}}$$

where *I* is the Morand’s *I*; *n* is the number of elements involved in the analysis; *x_i_* and *x_j_* represent the resource density of points *i* and *j*, respectively; *w_ij_* is the spatial weight coefficient of regions *i* and *j*, which reflects the spatial relationship between regions *i* and *j*, and if the regions are adjacent to each other, *w_ij_* = 1, otherwise, *w_ij_* = 0; *x* is the mean value of all elements; *E*(*I*) and *VAR*(*I*) denote the expectation and variance of Moran’s *I*, respectively. The value of *I* ranges from −1 to 1 to indicate whether the null hypothesis of a random distribution of resources within the study area is rejected [49]. When the value of *I* is greater than 0, this indicates positive correlation, and a value lower than 0 indicates negative correlation. The larger the absolute value, the higher the autocorrelation degree of spatial distribution, indicating that the distribution of resources presents the phenomenon of agglomeration, and the smaller the absolute value, the lower the autocorrelation degree of spatial distribution. When the spatial distribution exhibits a dispersed pattern (*I* = 0 or close to 0), it indicates no spatial autocorrelation in neighboring regions, suggesting a random resource distribution [50]. The *Z*-score and *p*-value are returned in the calculation of Moran’s *I*, where the *Z*-score is a multiple of the standard deviation of the *I* value. When the *Z*-score is negative, it indicates that the distribution of the element at a given distance is discrete, and when the *Z*-score is positive and large, it indicates that the element is distributed in a clustered manner. The *p*-value provides a test of significance for statistical results, indicating the probability that the sample spatial pattern includes some random distribution. A low *p*-value (typically <0.05) suggests a statistically significant spatial pattern, while a high *p*-value implies a random distribution [50]. 

Moran’s *I* was operated using the space function of GeoDa 1.14.0 software (https://geodacenter.github.io/download.html, accessed on 15 May 2025) for 99,999 randomized permutations.

### 2.3. Incremental Spatial Autocorrelation

The incremental spatial autocorrelation measures the spatial autocorrelation of a range of distances and selectively creates line plots of the *Z*-scores corresponding to these distances. The *Z*-score reflects the degree of spatial clustering [51], with statistically significant peaks indicating the distances that promote the clustering of spatial processes in the most pronounced way [52]. The peaks of these distances are usually the appropriate values for tools with a “Distance Range” or “Distance Radius” parameter. In this paper, the incremental spatial autocorrelation method was used to reveal the optimal distance threshold for the spatial analysis of the Japanese sardine population, and if multiple peaks existed, the distance corresponding to the first significance peak was selected as the distance parameter for the local spatial autocorrelation analysis in this study [53]. The calculation formula is as follows [54]:

(3) $$I\left(d\right)=\frac{n\sum_{i=1}^{n}\sum_{j=1}^{n}w_{ij}(d)\left(x_{i}-x\right)\left(x_{j}-x\right)}{\sum_{i=1}^{n}\sum_{j=1}^{n}w_{ij}(d)\sum_{i=1}^{n}{\left(x_{i}-x\right)}^{2}}$$

where *d* is the spatial interval. When *d* = 1 it indicates that the elements are adjacent, and *d* = 2 indicates that they are adjacent to elements with an interval of 1. Based on this, iterative calculations were performed for a set series of gradually increasing *d* values to obtain the Moran’s *I* index corresponding to different *d* values [55].

### 2.4. Local Spatial Autocorrelation

Global spatial autocorrelation describes the degree of autocorrelation in the distribution of attributes across the study area, but cannot effectively express the level of spatial autocorrelation between different spatial units in the study area and neighboring regions. The local spatial autocorrelation (hotspot analysis) can reveal the spatial clustering characteristics of attributes in the local area, identify the spatial clustering of high values (hotspots) and low values (coldspots) that are statistically significant, and be used to obtain the locations of areas of high (or low) abundance in fishing grounds that are spatially clustered [54]. The formula is calculated as follows:

(4) $${Gi}^{*}=\frac{\sum_{j=1}^{n}w_{i,j}x_{j}-X\sum_{j=1}^{n}w_{i,j}}{s\times \sqrt{n\left(\sum_{j=1}^{n}w_{i,j}^{2}-{\left(\sum_{j=1}^{n}w_{i,j}\right)}^{2}\right)/\left(n-1\right)}}$$

where *x_j_* is the attribute value of element *j*, *w_i,j_* denotes the spatial weight between elements *i* and *j* (1 for adjacent, 0 for non-adjacent), *n* is the total number of sample points, and *x* and *S* are the mean and the standard deviation, respectively. *G_i_* statistics return the *G_i_Z*-Score and *p*-value, whose significance is similar to that of the *Z*-Score and *p*-value returned by global spatial autocorrelation. When the *G_i_Z*-Score > 2.58 (or <−2.58) times the standard deviation, *p* < 0.01, the elements are spatial hotspots (or coldspots), and when the *G_i_Z*-Score is 1.96~2.58 (or −2.58~−1.96) times the standard deviation, *p* > 0.05, the resources may have a certain distribution of hotspots (or coldspots), and at this time, the possibility of random distribution is not excluded. When the *G_i_Z*-Score is 1.65~1.96 (or −1.96~−1.65) times the standard deviation, *p* > 0.01, the resources are very likely to be randomly distributed, and when the *G_i_Z*-Score is −1.65~1.65 times the standard deviation, the resources are randomly distributed in the study area [36].

### 2.5. Centre of Gravity Migration Trajectories and Standard Deviation Ellipse Analysis

The standard deviation ellipse is a spatial statistical method that quantifies the overall distribution characteristics and spatiotemporal evolution of research objects. Using the mean center, major/minor axes, and azimuth angle as parameters, it accurately describes spatial centrality in terms of direction, shape, and distribution [56]. This model effectively illustrates fishery change patterns [57] and marine population distribution variability [58]. The formula is calculated as follows:

(5) $$X_{w}=\frac{\sum_{i=1}^{n}(C_{i}\times X_{i})}{\sum_{i=1}^{n}C_{i}};\;Y_{w}=\frac{\sum_{i=1}^{n}(C_{i}\times Y_{i})}{\sum_{i=1}^{n}C_{i}}$$

(6) $$\tan\theta =\frac{\left(\sum_{i=1}^{n}{w_{i}}^{2}{{\tilde{x}}_{i}}^{2}-\sum_{i=1}^{n}{w_{i}}^{2}{{\tilde{y}}_{i}}^{2}\right)+\sqrt{\sum_{i=1}^{n}{w_{i}}^{2}{{\tilde{x}}_{i}}^{2}-\sum_{i=1}^{n}{w_{i}}^{2}{{\tilde{y}}_{i}}^{2}-4\sum_{i=1}^{n}{w_{i}}^{2}{{\tilde{x}}_{i}}^{2}{{\tilde{y}}_{i}}^{2}})}{2\sum_{i=1}^{n}{w_{i}}^{2}{\tilde{x}}_{i}{\tilde{y}}_{i}}$$

(7) $$\delta_{x}=\sqrt{\frac{\sum_{i=1}^{n}{\left(w_{i}{\tilde{x}}_{i}\cos\theta -w_{i}y_{i}\sin\theta \right)}^{2}}{\sum_{i=1}^{n}{w_{i}}^{2}}}$$

(8) $$\delta_{y}=\sqrt{\frac{\sum_{i=1}^{n}{\left(w_{i}{\tilde{x}}_{i}\sin\theta -w_{i}y_{i}\cos\theta \right)}^{2}}{\sum_{i=1}^{n}{w_{i}}^{2}}}$$

where *X_w_* and *Y_w_* denote the coordinates of the center of gravity of the fishing ground, *C_i_* is the yield of net *i*, *X_i_* is the latitude of net *i*, *Y_i_* is the longitude of net *i*, *n* is the total number of nets in the statistical period, *θ* is the ellipse azimuth, *w_i_* is the weight of the study unit, *x_i_* and *y_i_* are the center coordinates of each study area, ${\tilde{x}}_{i}$ and ${\tilde{y}}_{i}$ represent the deviation of the center coordinates of each study unit from the ellipse center of gravity coordinates, and *δ_x_* and *δ_y_* denote the standard deviation along the x-axis and y-axis, respectively. In the standard deviation ellipse analysis, the ellipse size was chosen to be 1 times the standard deviation.

### 2.6. Generalized Additive Model (GAM)

In this study, the GAM was used to analyze the spatial distribution of Japanese sardine in the high seas of NWPO. The CPUE of Japanese sardine was considered as the response variable, while the year, month, longitude, latitude, SST, Chl–a, SSS, and SSH were considered as the spatial and temporal explanatory variables. The GAM was constructed as follows:

log(CPUE + 1) ~ s(year) + s(month) + s(longitude) + s(latitude) + s(SST) + s(Chl–a) + s(SSS) + s(SSH) + s(Density) + ε (9)

where CPUE is the response variable; s(year) is the spatiotemporal explanatory variable year; s(month) is the spatiotemporal explanatory variable month; s(longitude) is the spatiotemporal explanatory variable longitude; s(latitude) is the spatiotemporal explanatory variable latitude; s(SST) is the environmental explanatory variable sea surface temperature; s(Chl–a) is the environmental explanatory variable chlorophyll a; s(SSS) is the environmental explanatory variable sea surface salinity; s(SSH) is the environmental explanatory variable sea surface height; and ε is the random error term.

The variance inflation factor (VIF) was used to test the independence of the explanatory variables, and the results are shown in Table 1. The predictor variables demonstrated acceptable collinearity (VIF < 5), consistent with the established diagnostic thresholds for regression models. This conservative criterion minimizes Type I errors while maintaining model parsimony, as justified by Monte Carlo simulations in ecological studies [59].

The model utilized a stepwise regression framework to select predictors by optimizing the Akaike Information Criterion (AIC) [60] and the bias explanation rate [61]. Improved model fit was indicated by lower AIC values and a higher bias explanation rate [62]. The model was implemented using the R package mgcv.

## 3. Results

### 3.1. Changes in Catches

**Figure 2:**
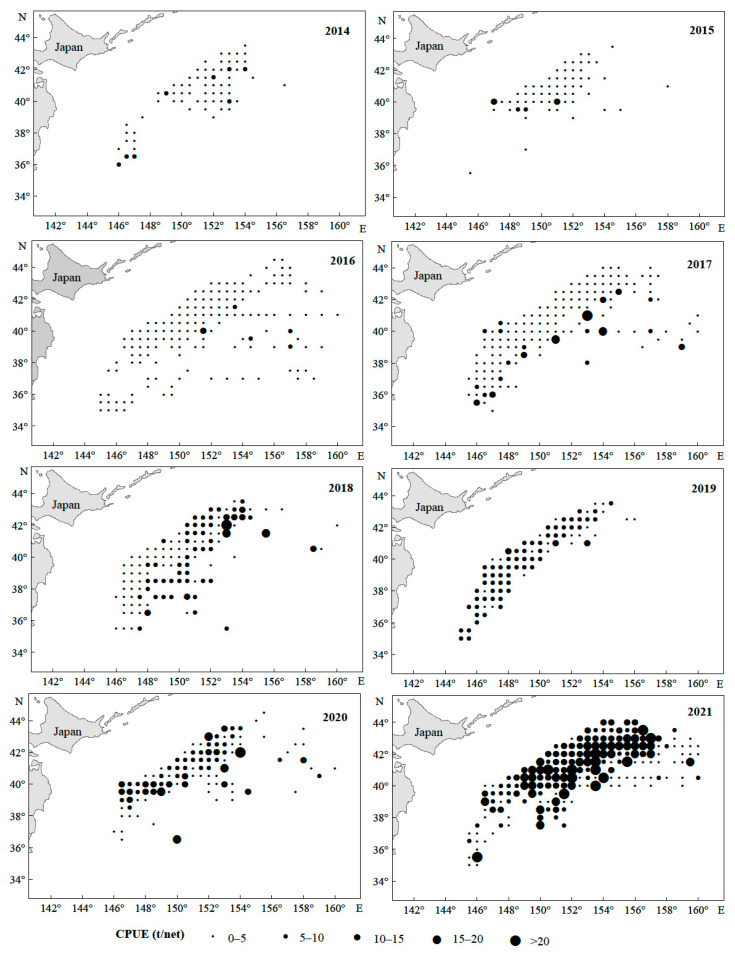
The spatiotemporal distribution of Japanese sardine CPUE in the NWPO from 2014 to 2021.

**Figure 3:**
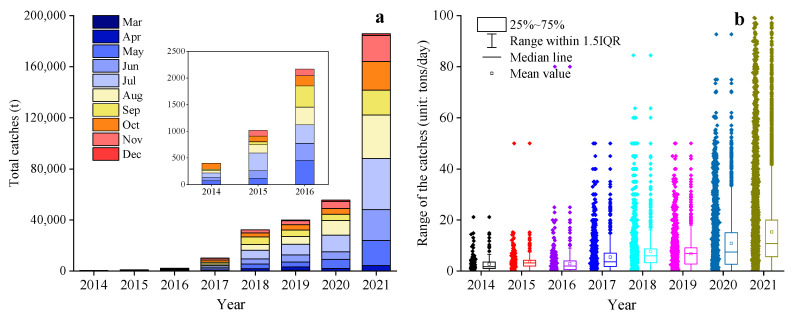
Changes in the yearly catches (**a**) and single-day catches (**b**) in the NWPO from 2014 to 2021.

**Figure 4:**
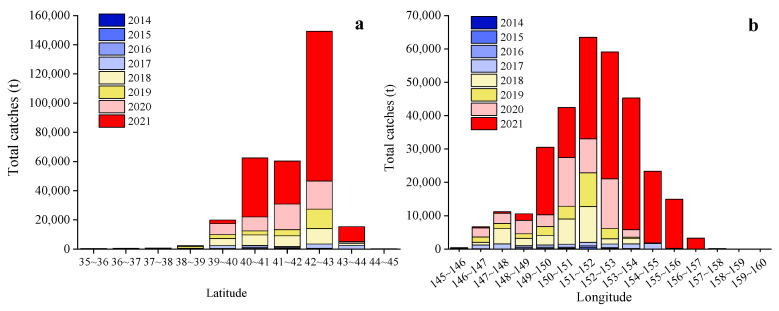
The variation of catches in the NWPO with latitude (**a**) and longitude (**b**) from 2014 to 2021.

From 2014 to 2021, the Japanese sardine fishery in the NWPO was distributed between 35–45° N and 145–160° E, primarily concentrated along the EEZs of Japan and Russia. The proportion of areas with a CPUE greater than 10 gradually increased over time: regions with a CPUE below 10 predominated from 2014 to 2017, whereas those exceeding 10 became dominant from 2018 to 2021, peaking in 2021 (Figure 2). The main fishing season occurred annually from April to November. Total catches rose steadily between 2014 and 2021, reaching a maximum of 185,788 tons in 2021 (Figure 3a). Single-day catches exhibited significant annual fluctuations but generally increased, except for slight declines in 2016 and 2019 (Figure 3b). Spatially, high-catch areas spanned a broader longitudinal range (149–155° E) than latitudinal range (40–43° N), with core zones clustered around 40–43° N and 149–155° E (Figure 4).

### 3.2. Fishing Centers of Gravity and Distribution Patterns

**Figure 5:**
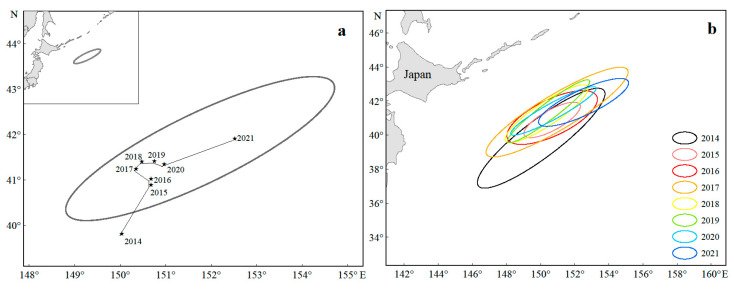
Changes in the center of gravity (**a**) and SDE of the fishing grounds (**b**) from 2014 to 2021.

**Figure 6:**
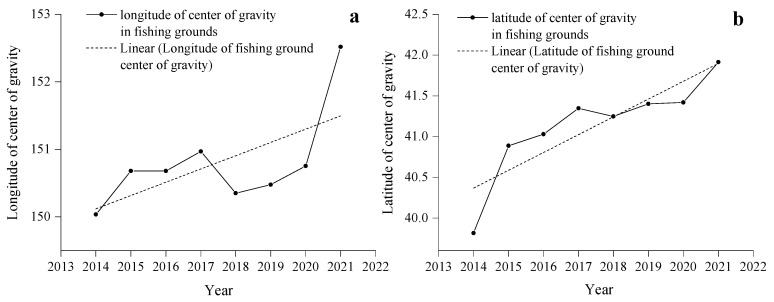
Variations in the longitude (**a**) and latitude (**b**) of the Japanese sardine fishing ground center of gravity from 2014 to 2021.

**Figure 7:**
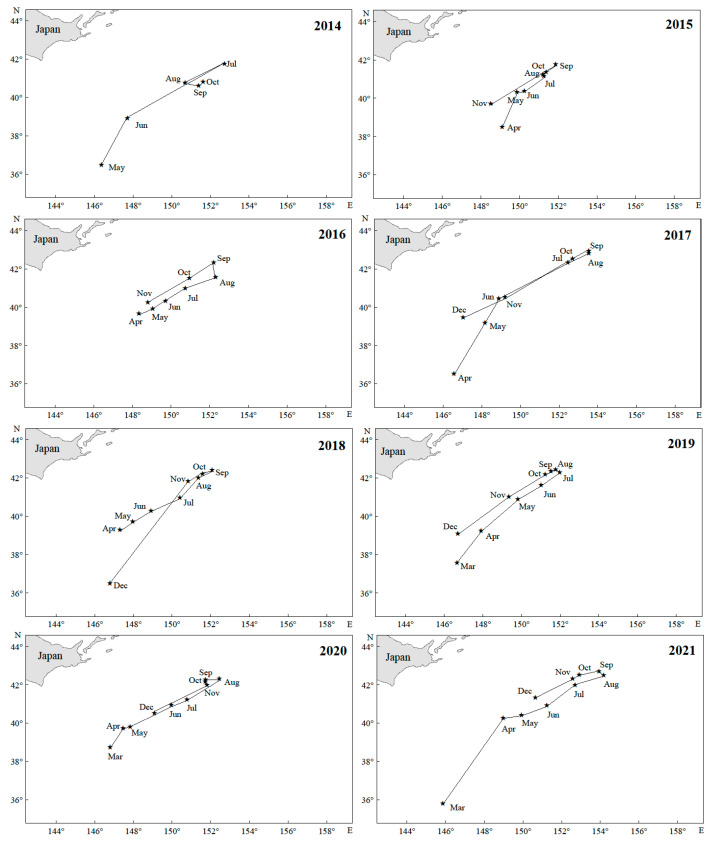
Monthly changes in the gravity center of the fishing grounds in the NWPO from 2014 to 2021.

From 2014 to 2021, the center of gravity of the Japanese sardine fishing grounds in the NWPO shifted, moving northwest during 2014–2018 and northeast from 2019–2021, with an overall northeastward trend (Figure 5a). Longitudinal changes occurred faster and spanned a wider range (150.03–152.52° E) compared to latitudinal shifts (39.81–41.91° N) (Figure 6). Seasonal patterns showed consistent annual cycles: the fishing grounds migrated northeastward from March (or April), peaked in August (or September), then retreated southwestward, ending in November (or December) at the southwesternmost point—still northeast of the initial position (Figure 7).

The spatial distribution exhibited a stable southwest–northeast directional pattern (Figure 5a,b), with minimal annual variation in azimuth angles (52.70–64.79°, Table 1), indicating high directional consistency. The annual expansion of fishing areas fluctuated, peaking in 2014 and reaching a minimum in 2015. High standard deviation ellipse oblateness values (2.73–6.20) further confirmed strong centripetal aggregation of sardine populations (Table 1).

### 3.3. Global Spatial Autocorrelation Analysis and General Statistics

From 2014 to 2021, Japanese sardine abundance data showed a positive skew (>0) and leptokurtic distributions (kurtosis > 3), indicating sharper peaks and higher vertical probability density compared to a normal distribution, with more low-abundance areas. High spatial variability (Cv > 0) further confirmed uneven abundance across locations (Table 2).

**Figure 8:**
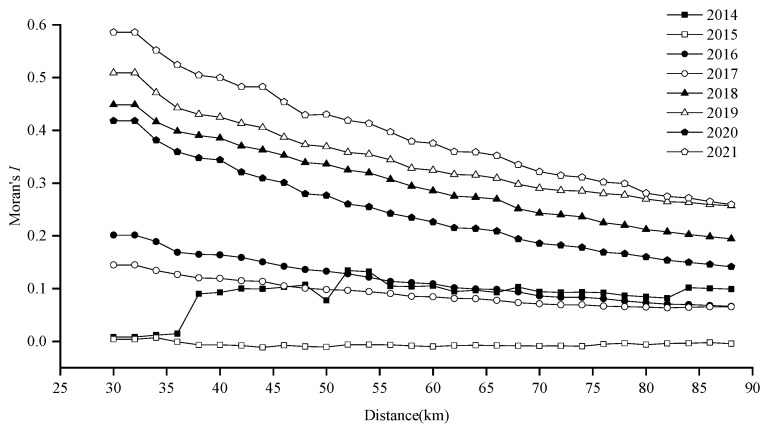
Results of incremental spatial autocorrelation analysis for Japanese sardine resource density in the NWPO from 2014 to 2021.

Global spatial autocorrelation analysis revealed significant annual clustering (positive Moran’s *I*, *Z*-score > 1.96, *p* < 0.0001), except in 2015 (Moran’s *I* ≈ 0, *p* > 0.05), when resources were dispersed. As distance increased, spatial autocorrelation generally weakened (Figure 8).

**Figure 9:**
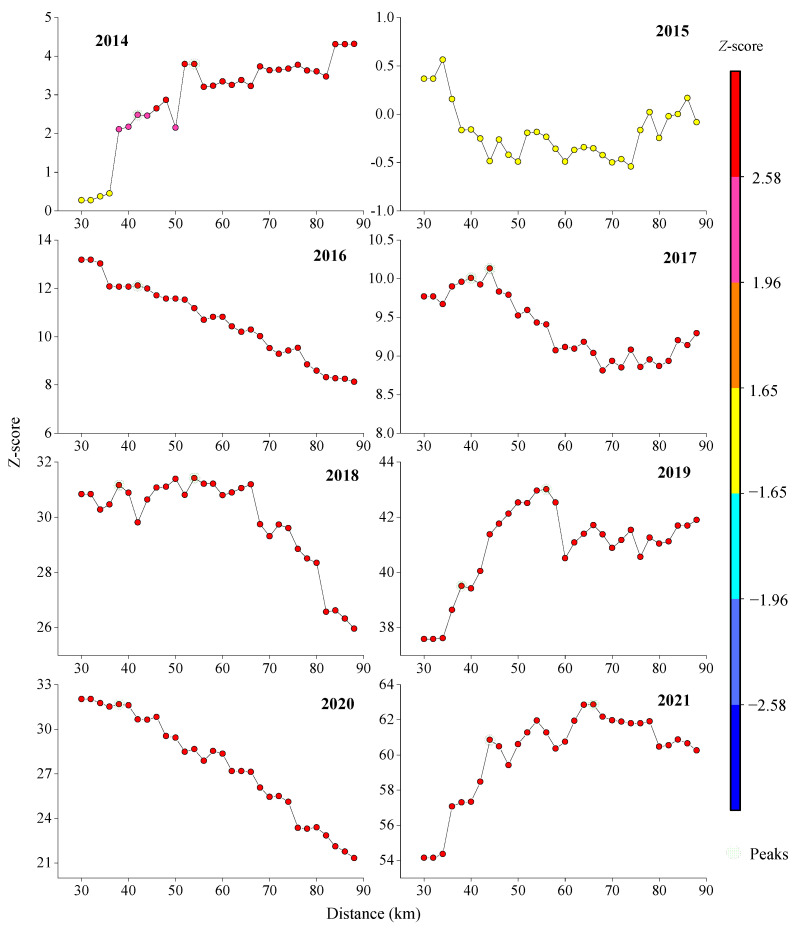
The incremental spatial autocorrelation model index displaying the resource density of the Japanese sardine in the NWPO from 2014 to 2021.

*Z*-scores exceeded 2.58 (*p* < 0.01) in all years except 2015, confirming strong aggregation at the 99% confidence level. Optimal spatial autocorrelation thresholds varied annually (38–44 km), with peak *Z*-scores at 42 km (2014, 2016–2021) and 44 km (2021), indicating significant clustering at these scales (Figure 9).

### 3.4. Distribution of Coldspots and Hotspots 

**Figure 10:**
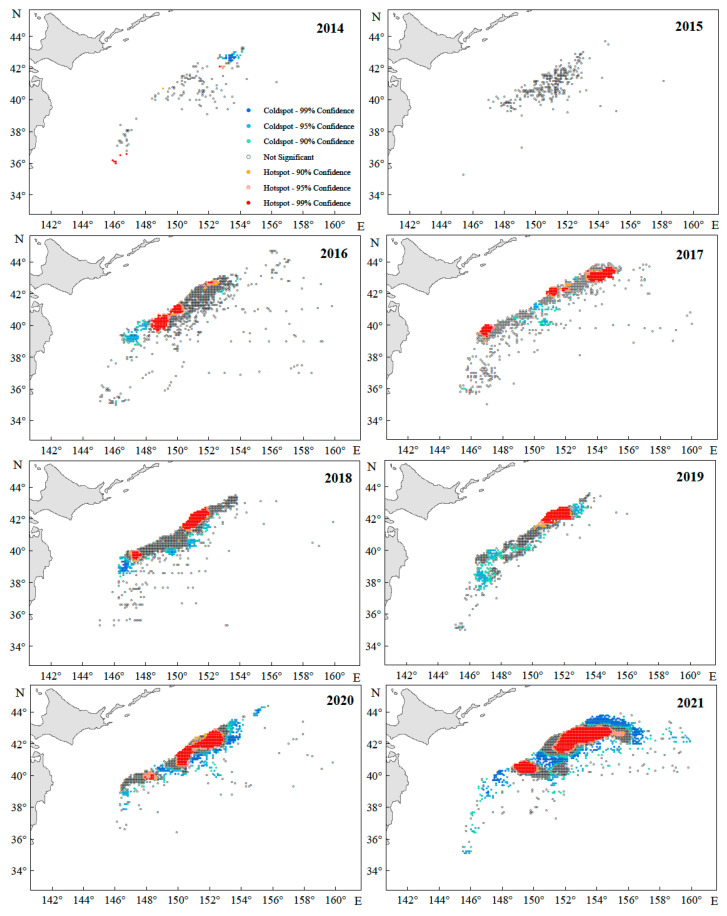
The annual catch hotspots of the Japanese sardine in the NWPO.

Overall, hotspots and coldspots of Japanese sardine resources exhibited interannual spatial clustering with significant temporal variability (Figure 10). In 2015, resources showed a random distribution, aligning with global and incremental spatial autocorrelation analyses. Hotspots displayed a northeastward migration trend over time, mirroring the shifting center of gravity of the fishing grounds. These hotspots formed multiple clustered patches, with their core range narrowing northward from 40–42° N (2016) to 42–44° N (2017–2021). Although annual fluctuations occurred in hotspot/coldspot coverage, hotspots progressively occupied a larger proportion of the fishing area. 

### 3.5. GAM Analysis 

#### 3.5.1. GAM Test 

The VIF test results are presented in Table 3. Since all factors have VIF values below 5, all variables were included in the model constructed for this study.

**Figure 11:**
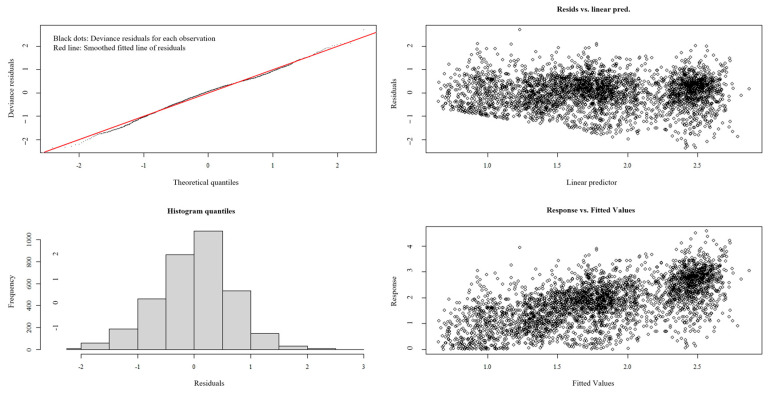
The diagnostic analysis of residuals.

As summarized in Table 4, the optimal GAM demonstrated a robust performance, with an AIC of 9861.046, 41.4% deviance explained, and an R^2^ value of 0.411. To validate the model’s generalizability, residual diagnostics were rigorously applied. Residual analysis revealed a lack of systematic patterns or heteroscedasticity, alongside a stable effective degrees of freedom (edf) trajectory and declining AIC, consistent with appropriate regularization (Figure 11). These results collectively demonstrate that the GAM, optimized through iterative refinement, avoids overfitting and reliably captures underlying data structures.

The statistical significance of variables in the final optimized GAM is presented in Table 5. Results from the F-test analysis revealed that all predictors, including interaction terms, demonstrated statistically significant associations with length measurements (*p* < 0.01).

A stepwise regression procedure, guided by AIC, VIF, and R^2^, was implemented to identify optimal predictors. Year, month, longitude, latitude, SST, chl–a, SSS, and SSH were selected as covariates, with length operationalized as the response variable. The generalized additive model (GAM) was formulated as Formula (9).

#### 3.5.2. Distribution of CPUE Under Different Factors 

**Figure 12:**
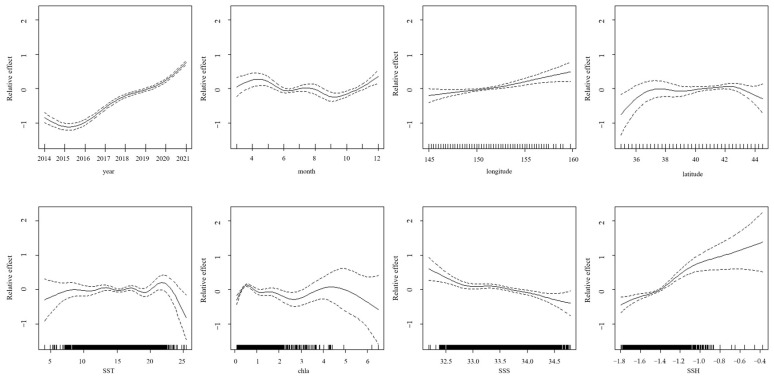
The impact of each explanatory variable on the CPUE of the Japanese sardine in the NWPO (The dashed lines in the diagram represent the 95% confidence interval, and the solid lines represent the actual values).

The GAM analysis of the Japanese sardine CPUE in the high seas of the NWPO revealed complex relationships with multiple environmental and spatiotemporal factors (Figure 12). From 2014 to 2021, the CPUE exhibited an initial decline followed by a gradual recovery, indicating interannual variability. In June and September, the CPUE had a significantly lower value. Spatial patterns suggest a positive correlation with longitude (higher CPUE in eastern areas). The CPUE remained stable when the latitude was in the range of 37° N to 43° N, and declined beyond this range. Environmental regulators showed differential effects: the CPUE remained stable within SST thresholds (10–20 °C) but declined beyond this thermal range. Chl–a concentrations > 5 mg/m^3^ corresponded to a diminishing CPUE, whereas SSS displayed inverse correlations and SSH exhibited positive associations with the CPUE. 

## 4. Discussion

### 4.1. Geographical Distribution and Temporal Variation of Catches 

Fishing is primarily a profit-driven activity [63], where maximizing profits often takes priority over conserving resources or protecting marine areas. While fishing operations directly influence catch locations, the underlying distribution patterns depend on fish stock availability. These stocks are affected by factors like climate, sea temperature, and food supply. With advances in forecasting and exploration technology [64], modern fishing can target specific areas more accurately, making catch distributions reflect actual fish population dynamics.

This study found that Japanese sardine fishing grounds are geographically concentrated along Japan’s marine EEZ baseline (Figure 1). The nutrient-rich waters in this area, created by the convergence of the warm Oyashio and cold Kuroshio currents [26,65], significantly influence Northwest Pacific Ocean (NWPO) fishery resource distribution [66,67], explaining the concentration of sardine fisheries. Japanese sardines in the NWPO primarily inhabit 40–43° N and 149–155° E, overlapping with chub mackerel distribution. This spatial overlap suggests either shared habitat preferences or a predator–prey relationship between the species. 

Ma et al. [8] found that since 2010, the intensified Siberian high-pressure system has created favorable climate conditions, combined with reduced fishing pressure, leading to Japanese sardine recovery in the NPWO. This likely explains their catch increases from 2014–2021. Japanese sardine and chub mackerel biomass in the NWPO show an inverse relationship [5]. Recent chub mackerel catch declines [5,42,66] may have further boosted sardine catches through species competition [68,69]. Despite consecutive catch growth since 2014 (Figure 3), fishing vessel numbers did not correlate—2017 had more vessels than 2019–2020 [67], yet lower catches. This suggests that fishing effort is not the primary catch determinant [42]. Japanese sardines exhibit strong resilience to fishing pressure [20,70], especially with current stock growth [25,26], indicating high exploitation potential. However, dynamic factors like density dependence, fishing effort, and climate change pose management challenges [8].

### 4.2. Analysis of Changes in Fishing Ground 

The center of gravity of fishing grounds is a key metric in fishery science that is used to track fishing ground shifts and identify core fishing areas [71]. From 2014 to 2021, the Japanese sardine fishing grounds shifted northeastward (Figure 5), moving 2.49° east and 2.10° north (Figure 6). This movement reflects multiple factors including temperature anomalies, food availability, and ecosystem changes [72]. Japanese sardines show particular sensitivity to environmental shifts [73], with climate warming likely driving their northward migration. Our study confirms their preference for SST between 10 and 20 °C (Figure 12), which aligns with the findings of Yang et al. [74]. SSH and SST are recognized as the most critical factors influencing Japanese sardine habitat changes, contributing more significantly to these shifts than chla and SSS [45]. Japanese sardines, as SST-sensitive pelagic forage fish, rely on zooplankton prey whose distribution is modulated by SSH [45]. This dual dependency on thermal and hydrodynamic drivers underpins SSH-SST synergism in shaping their habitat niches. Research indicates that marine species are responding to warming through poleward migration [75]. This northward shift has become a widespread pattern among NWPO fish stocks [5]. Notably, sardine populations in the western and eastern Pacific boundary current systems exhibit diametrically opposed responses to decadal-scale sea temperature anomalies, yet the mechanisms driving these contrasting dynamics remain unresolved [76].

The population dynamics of small pelagic species are strongly governed by oceanographic drivers [77,78]. Japanese sardine populations exhibit a seasonal migration pattern, moving northeastward from March (or April) before returning southwest in September, ultimately settling northeast of their starting position (Figure 7). These findings demonstrate that the spatial extent of suitable habitats for Japanese sardines exhibits significant dynamic variations, driven by spatiotemporal changes in the marine environment. Environmental variability drives monthly shifts in the distribution range of suitable habitats and the monthly geometric centers of habitat suitability index values [45]. This movement reflects their ecological behavior, primarily driven by temperature changes and daylight duration [79]. The Kuroshio Current plays a crucial role in this migration, particularly in transporting larvae to the Kuroshio Extension during April–June for growth [80]. Water mass dynamics along the Kuroshio axis also significantly influence sardine population replenishment [27]. 

The Japanese sardine fishing grounds in the high seas of the NWPO exhibit a distinct southwest–northeast distribution, with strong fishery aggregation. From 2014 to 2021, the fishery expanded northeastward while showing a shrinking trend in its expansion area (Figure 5 and Figure 6). These findings confirm the gradual northward shift of Japanese sardine stocks and reflect improved accuracy in fishery forecasting, fish exploration, and fishing technology. Zhao et al. [32] noted that while fleet operations influence fishing ground expansion, fish population dynamics play a more critical role. Fishing ground changes ultimately depend on fish aggregation patterns, which are shaped by their ecological behavior.

### 4.3. Spatial Autocorrelation Analysis 

The results demonstrate that Japanese sardine fishing grounds exhibit distinct spatial autocorrelation. All study years except 2015 displayed varying levels of spatially positive autocorrelation, reflecting significant aggregation patterns (Table 2). Spatial autocorrelation consistently decreased as threshold distance increased (Figure 8). This pattern may help reduce intraspecific competition caused by high fish densities [81], maintaining normal ecological behaviors through spatial distribution adjustments [32]. Additionally, autocorrelation was lower in low-catch years compared to high-catch years. Notably, sardine autocorrelation showed an inverse relationship with Japanese mackerel catches, suggesting that interspecific competition or predator–prey dynamics may influence their spatial patterns. We propose three key factors governing fishery resource autocorrelation: intraspecific competition [32,36], interspecific competition, and predator–prey relationships. The hotspot analysis employed standard null hypothesis testing to identify statistically significant clusters. 

The distribution of aquatic organisms generally conforms to the characteristics of a spherical semivariance function, the spatial dependence of sample points tends to decrease gradually with increases in the distance between the sample points [82], and the coldspots and hotspots have strong clustering characteristics. In this paper, we found that the Japanese sardine fishing grounds had obvious coldspots and hotspots, and there were large differences in the distribution areas and ranges of coldspots and hotspots between years. The hotspot areas generally consisted of multiple patches, and their spatial and temporal variability characteristics can be corroborated with the center of gravity of the fishing grounds [83]. Thus, similar to the northward shift of the center of gravity of the fishing grounds, the increase in seawater temperature may be the main factor influencing the characteristics of the regional distribution of coldspots and hotspots. It was reported that coldspots and hotspots in the fishery are areas subjected to high-frequency fishing, and the difference lies in the low yield of the former and the high yield of the latter, while for randomly distributed areas, the case may be that the frequency of operation and the yield are low, or it may be that the spatial autocorrelation is low [38]. It is important to mention that both coldspots and hotspots are both the central fishing grounds of the fishery, and changes in coldspots and hotspots often reflect changes in the characteristics of the central fishing grounds.

## 5. Conclusions

This study analyzed the spatiotemporal distribution characteristics of Japanese sardine fishing grounds in the high seas of the NWPO. The findings show that sardine catches increased annually from 2014 to 2021, with accelerated growth in 2021. The fishing ground’s center gradually shifted northeastward, exhibiting a distinct southwest–northeast distribution pattern with strong directional aggregation. The resource distribution demonstrated significant spatial clustering, showing global spatial autocorrelation in abundance. The Japanese sardine abundance displayed clear coldspot and hotspot patterns, with hotspots showing northeastward movement tendencies. Additionally, sardine distribution is influenced by multiple factors including temporal, spatial, and environmental variables.

## Figures and Tables

**Table 1:** The SDE parameters of the Japanese sardine resource density in the high seas of the NWPO.

Year	Rotation	Major Axis	Short Axis	Oblateness
2014	52.70	4.63	1.09	4.26
2015	61.02	1.86	0.56	3.14
2016	64.30	2.89	1.06	2.73
2017	58.98	4.82	1.02	4.69
2018	56.42	2.88	0.68	4.26
2019	52.89	3.00	0.48	6.20
2020	61.25	2.84	0.47	6.02
2021	64.79	2.90	0.74	3.89

**Table 2:** The global spatial autocorrelation parameters for the annual catch of Japanese sardine.

Year	Mean	SD	Skewness	Kurtosis
2014	3.01	3.57	2.75	8.72
2015	3.72	4.04	6.15	64.34
2016	2.81	4.04	10.38	181.64
2017	5.59	6.1	2.74	10.86
2018	7.67	8.35	3.255	13.953
2019	6.82	5.62	2.12	9.55
2020	10.99	11.19	1.98	6.33
2021	15.34	14.26	2.08	5.84

**Table 3:** The variable VIF testing results.

Variable	Year	Month	Longitude	Latitude	SST	Chl–a	SSS	SSH
VIF value	3.337	1.654	3.179	3.183	1.782	1.484	4.788	4.998

**Table 4:** GAM selection based on AIC.

GAM	R^2^	AIC	Explanation Rate (%)
log(CPUE + 1)~*s*(year)	0.361	6989.464	36.1
log(CPUE + 1)~*s*(year) + *s*(month)	0.373	6929.975	37.6
log(CPUE + 1)~*s*(year) + *s*(month) + *s*(longitude)	0.378	6904.907	38.1
log(CPUE + 1)~*s*(year) + *s*(month) + *s*(longitude) + *s*(latitude)	0.379	6901.542	38.2
log(CPUE + 1)~*s*(year) + *s*(month) + *s*(longitude) + *s*(latitude) + s(SST)	0.384	6885.366	38.8
log(CPUE + 1)~*s*(year) + *s*(month) + *s*(longitude) + *s*(latitude) + s(SST) + s(chl–a)	0.395	6833.707	40
log(CPUE + 1)~*s*(year) + *s*(month) + *s*(longitude) + *s*(latitude) + s(SST) + s(chl–a) + s(SSS)	0.396	6830.116	40.3
log(CPUE + 1)~*s*(year) + *s*(month) + *s*(longitude) + *s*(latitude) + s(SST) + s(chl–a) + s(SSS) + s(SSH)	0.408	6773.745	41.6

**Table 5:** ANOVA of the optimal GAM.

Parameter	df	F	*p* Value
Year	4	476.3	2 × 10^−16^
Month	3.926	5.807	9.35 × 10^−5^
Longitude	3.987	35.8	2 × 10^−16^
Latitude	1.907	28.51	2 × 10^−16^
SST	3.924	8.005	1.36 × 10^−5^
chl–a	3.992	6.25	2 × 10^−16^
SSS	3.424	14.49	2 × 10^−16^
SSH	3.982	119.6	2 × 10^−16^

## Data Availability

The original contributions presented in the study are included in the article, and further inquiries can be directed toward the corresponding authors.
